# A chronic wound model to investigate skin cellular senescence

**DOI:** 10.18632/aging.204667

**Published:** 2023-04-21

**Authors:** Saranya P. Wyles, Parisa Dashti, Tamar Pirtskhalava, Burak Tekin, Christina Inman, Lilian Sales Gomez, Anthony B. Lagnado, Larissa Prata, Diana Jurk, João F. Passos, Tamar Tchkonia, James L. Kirkland

**Affiliations:** 1 Department of Dermatology, Mayo Clinic, Rochester, MN 55905, USA; 2 Department of Physiology and Biomedical Engineering, Mayo Clinic, Rochester, MN 55905, USA; 3 Department of Laboratory Medicine and Pathology, Mayo Clinic, Rochester, MN 55905, USA; 4 Department of Medicine, Division of General Internal Medicine, Mayo Clinic, Rochester, MN 55905, USA

**Keywords:** wound healing, cellular senescence, chronic wound, re-epithelization, skin

## Abstract

Wound healing is an essential physiological process for restoring normal skin structure and function post-injury. The role of cellular senescence, an essentially irreversible cell cycle state in response to damaging stimuli, has emerged as a critical mechanism in wound remodeling. Transiently-induced senescence during tissue remodeling has been shown to be beneficial in the acute wound healing phase. In contrast, persistent senescence, as observed in chronic wounds, contributes to delayed closure. Herein we describe a chronic wound murine model and its cellular senescence profile, including the senescence-associated secretory phenotype.

## INTRODUCTION

Skin wound healing is an essential and evolutionarily conserved mechanism that benefits multiple species including mammals. Because of the role of skin as a physical, chemical, and bacterial barrier, skin wound healing can serve as a surrogate marker for skin aging and overall skin health [1]. Wounds can be classified based on various factors, including wound duration as an acute versus chronic wound [2]. Chronic wounds, which include diabetic ulcers, vascular ulcers, and pressure ulcers, among others, are defined as a skin barrier defect that persists beyond three months despite standard-of-care, contributing to healthcare burden and morbidity [3]. Despite a complex series of cellular signaling and behavioral events that ensure skin barrier closure in acute wounds, minor disruptions rarely cause issues in wound healing due to high levels of cell redundancy and compensatory mechanisms [4, 5]. For instance, the ablation of specific subsets of hair follicle stem cells [6], MMPs [7], fibroblast growth factors [8], TGF-α [9], and VEGFR2 [10] each individually fail to substantially hinder wound closure. Hence, understanding molecular and pathological causes of chronic wounds, which primarily affect elderly and diabetic populations, is crucial.

An emerging area in studying wound healing, particularly chronic wounds, is the role of cellular senescence. Cellular senescence is a cell fate that involves essentially irreversible replicative arrest, apoptosis resistance, often amplified protein synthesis, metabolic shifts with accentuated glycolysis, reduced fatty acid oxidation, increased reactive oxygen species generation, and acquisition of a senescence-related secretory phenotype [11]. Senescent cells have been shown to have a causal role in aging and age-related disorders [12–14]. In fact, a combination of stimuli (i.e., tissue injury) can trigger cells to enter a state marked by significant chromatin and secretome alterations, increased expression of the cell cycle inhibitor *p16^INK4a^*, replicative arrest, and apoptosis resistance [12, 15]. Furthermore, senescent cells can produce senescence-associated secretory phenotype (SASP) factors, which can include pro-inflammatory cytokines, chemokines, and proteins that degrade the extracellular matrix (ECM) [16–19]. As senescent cells accumulate during aging in the skin, the presence of even low numbers of senescent cells can be sufficient to cause tissue dysfunction [20–22].

Herein we hypothesize that persistent senescent cell accumulation contributes to delayed healing in chronic wounds. This study presents a novel oxidative stress-induced chronic murine wound mouse model in which there is capacity to target aberrant senescent cell expression. Pharmacological manipulation of oxidative stress can influence wound healing and result in delayed wound closure [23, 24], which offers the opportunity to characterize cellular senescence in late stages of wound healing. The molecular and histological profiles of senescent cells in the epidermis and dermis demonstrate the adverse influence of SASP factors in the chronic wound bed, a new avenue for root-cause, targeted therapeutic interventions.

## MATERIALS AND METHODS

### Animals, diet, and cohorts

**Figure 1:**
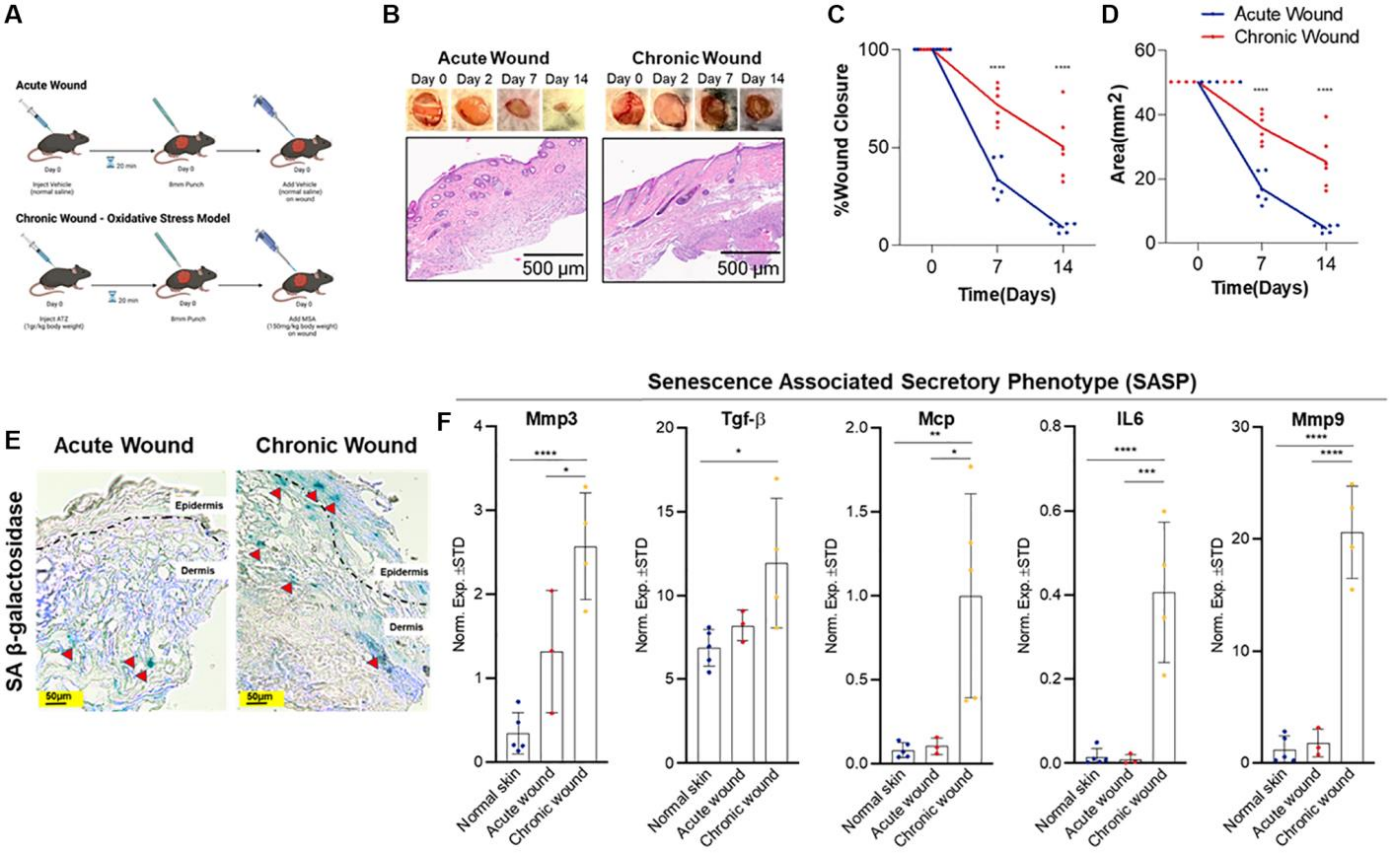
**Oxidative stress-induced wounding results in a chronic wound with increased SA-β-gal expression and SASP burden.** (**A**) Study design: an acute wound model (top) created with skin punch biopsy and vehicle (normal saline) application and a chronic wound model (bottom) created with localized oxidative stress induced by intraperitoneal 3-amino-1,2,4-trizole (ATZ; 1 g/kg) prior to wounding and topical mercaptosuccinic acid (MSA; 150 mg/kg) after wounding in wild-type C57BL/6 J mice (20-weeks-old). (**B**) Representative images of wound healing and histological images from hematoxylin and eosin-stained sections of acute versus chronic wounds (*n* = 6 in each group at day 14), low power magnification. (**C**) Wound contracture assessment as a function of % wound closure. (**D**) Wound contracture assessment as a function of area (mm^2^). (**E**) SA-β-gal staining indicates presence of senescent cells (red arrows) in the epidermis and dermis 14-days post-wounding. (**F**) Relative expression of senescence and SASP markers in the skin after 14-days in normal skin, acute wounds, and chronic wounds. Measurements are expressed as mean ± SEM. Statistical analysis was performed using Student’s *t*-test; ^*^p < 0.05, ^**^p < 0.01, ^***^p < 0.001, ^****^p < 0.0001.

All mouse experiments were performed in accordance with protocols approved by the Institutional Animal Care and Use Committee (IACUC) at Mayo Clinic. Twenty-week-old wild-type C57BL/6 mice were obtained from the Jackson Laboratories and maintained in a pathogen-free facility at 23–24°C under a 12-hour light, 12-hour dark regimen with free access to normal chow diet (standard mouse diet with 20% protein, 5% fat (13.2% fat by calories), and 6% fiber; Lab Diet 5053, St. Louis, MO) and water. Localized oxidative stress was induced by using 3-amino-1,2,4-trizole (ATZ) and mercaptosuccinic acid (MSA), which inhibit catalase and glutathione peroxidase, respectively, as previously described [25]. ATZ (Sigma Aldrich, St. Louis, MO, USA) was injected intraperitoneally at 1g ATZ/kg of mouse weight in sterile PBS roughly 20 minutes prior to surgery. An 8 mm full-thickness excisional wound was made on back of each mouse after hair removal by shaving and application of depilatory lotion. MSA (Sigma Aldrich, St. Louis, MO, USA) was administered topically onto the wound site at 150 mg MSA/kg of mouse weight in sterile PBS and subsequently covered with 3M™ Tegaderm™ Film Dressing (Figure 1A). For analgesia, mice were treated with carprofen intraperitoneally at 10 mg/kg in sterile PBS prior to surgery and 6 hours post-surgery. Mice were housed individually after wounding.

### Wound imaging

Wound photography was performed using an Olympus TG-6 Digital Camera with LED Light Guide (Olympus, Shinjuku, Japan). Wounds were photographed at baseline, day 2, day 7, and day 14. Wound imaging from RNA *in situ* hybridization (ISH) was performed using a Nikon T1 microscope (Nikon, Japan). Background correction and intensity thresholding were defined using controls and applied to all samples using Advanced NIS Elements software (Nikon, Tokyo, Japan). A total of 4 to 5 sections/slide with the best tissue integrity were selected for counting, and merged images were exported to ImageJ FIJI. We applied a centralized grid of 125 × 125 mm, generating 15 fields/section. *p16^Ink4a+^* and *p21^Waf1/Cip1+^* cell counting markers were used to retrieve cell numbers in each square. A single channel for DAPI was exported to ImageJ and the same 125 × 125 μm grid was applied to count nuclei in each square slice.

### Quantitative real-time reverse transcriptase PCR (RT-qPCR)

Tissues were flash-frozen in liquid nitrogen and kept frozen until RNA extraction. Tissues were lysed using TRIzol Reagent (Invitrogen, Waltham, MA) and RNA was isolated using a Direct-zol RNA MiniPrep Kit (Zymo Research, Irvine, CA, USA) with a DNA digestion kit (Zymo Research, Irvine, CA, USA). Isolated RNA was reverse transcribed into cDNA using the SuperScript III First Strand Synthesis System (Invitrogen, Waltham, MA, USA). Gene expression was quantified using RT-qPCR in which each reaction was performed with 10 ng cDNA per 10 μl, a QuantiTect SYBR Green PCR Kit (Qiagen, Hilden, Germany), and the CFX384 Real-Time System machine (BioRad, Hercules, CA, USA). Transcript levels were quantified using the 2^ΔΔ^Ct method and normalized to the housekeeping gene β- actin using gene-specific primer sequences (Supplementary Table 1).

### Senescence-associated β-galactosidase (SA-β-gal) activity assay

Frozen tissues were cut into 7 μm sections and fixed immediately in 2% formaldehyde (F877-500 ml Sigma) + 0.2% glutaraldehyde (G5882-10 × 10 ml Sigma) in PBS for 10 minutes at room temperature and washed with PBS. Tissues were incubated in SA-β-gal activity solution, pH6.0 at 37°C for 16–18 hours (overnight), washed, stained with Hoechst dye, and kept in PBS until imaged with a fluorescence microscope (Nikon Eclipse Ti, Japan). Ten to 15 random fields were imaged per sample. SA-β-gal^+^ cells are expressed as a function of all nuclei in the fields [26].

### RNA *in situ* hybridization and histological assessment

Tissues were formalin-fixed and paraffin-embedded. RNA-ISH was performed using the RNAscope protocol from Advanced Cell Diagnostics, Inc. (Hayward, CA, USA). Paraffin sections were deparaffinized, rehydrated in graded ethanol, and then H_2_O_2_ was applied. Sections were processed as previously described [27]. A RNAscope 2.5 HD Reagent kit-RED was used for chromogenic labeling. Tissues were mounted using ProLong Gold Antifade Mountant with DAPI (Invitrogen, Waltham, MA, USA) [27]. Counts for *p16*^Ink4a^- and *p21^Waf1/Cip1^*-positive cells were calculated using Fiji-ImageJ software.

### Statistical analysis

GraphPad Prism 7.0 was used for statistical analysis. Two-tailed Student’s *t*-tests were used to estimate statistically significant differences between two groups. One-way analysis of variance (ANOVA) with Tukey’s *post hoc* comparison was used for multiple comparisons. Values are presented as mean ± SEM unless otherwise indicated, with *p* ≤ 0.05 considered to be significant.

### Data availability

The datasets generated during and/or analyzed during the current study are available from the corresponding author upon reasonable request.

## RESULTS

### Oxidative stress-induced wounding results in a chronic wound model that is distinct from an acute wound model

Wound healing time is determined by several metrics, including wound size, depth, location, age, and the presence of local and systemic disease. Chronic wounds fail to undergo an orderly sequence of repair to restore normal anatomy and function, whereas acute wounds progress through the wound healing phases in a stereotypic sequential fashion. We created an 8-mm wound in the acute versus chronic murine model and measured wound closure and wound area alteration through the 14-day time course (Figure 1B–1D). For the chronic wound model, oxidative stress was induced by treating the wounds with two inhibitors of antioxidant enzymes, catalase (inhibited by 3-Amino-1,2,4-triazole (ATZ) [28]) and glutathione peroxidase (inhibited by mercaptosuccinic acid (MSA) [29]), resulting in a chronic wound that shares similar features observed in human diabetic chronic wounds due to prolonged inflammation from oxidative stress [25]. Potential off-target effects from ATZ and MSA include decreased nitric oxide availability, vasoconstriction, and other sequalae from increased reactive oxygen species (ROS) [30, 31]. These effects of oxidative injury are limited given localized versus systemic delivery. Wound closure rate and wound size significantly decreased in acute wounds compared to oxidative stress-induced chronic wounds. Wound healing occurs in four overlapping phases: hemostasis, inflammation, proliferation, and remodeling [32]. Within minutes following injury, neutrophils bind to endothelium, initiating the inflammatory phase that causes bacterial phagocytosis, matrix protein degradation, and further neutrophil migration [33]. We considered a pathological criterion that indicates the degree of inflammation by examining sparse, moderate, and diffuse inflammation. We found diffuse and persistent inflammation in all chronic wounds in contrast to acute wounds, which exhibited minimal to no inflammation at day 14 (Figure 1B). The proliferation phase includes fibroplasia, granulation, epithelialization, and angiogenesis, which begin within 24 hours [34]. TGF-β stimulates keratinocytes to migrate from the wound edge through fibrin matrix to subsequently develop a network in the wound bed [35]. None of the acute wounds displayed epidermal hyperplasia at day 14, whereas most of the chronic wounds revealed subtle foci of epidermal hyperplasia (Figure 1B, 1D), suggesting minimal wound contraction, and confirming the histological difference between acute and chronic wounds. Blood vessel thickening was also detected in chronic wounds (Figure 1B). Therefore, we considered this oxidative stress-induced model to be reliable method that recapitulates physiologically relevant chronic wounds.

### Increased SA-β-gal and SASP burden in chronic wounds compared to acute wounds

SA-β-gal, a cellular senescence biomarker [36], has been used to evaluate elevated, pH-shifted β-galactosidase activity in senescent cells. The SA-β-gal assay in acute versus chronic wound samples indicates that SA-β-gal-positive cells are increased in both epidermis and dermis in the chronic wounds compared to relatively lower numbers of positive cells in the dermis of acute wounds (Figure 1E).

Senescent cells can have a complicated secretome that includes a variety of cytokines, chemokines, and proteases, among other factors [19, 37]. This SASP, also known as the senescence messaging secretome (SMS) [19], indicates senescent cells’ non-cell autonomous functioning and may explain their *in vivo* participation in chronic wound pathophysiology. MMPs play essential roles in all wound healing phases: during the inflammatory phase, they remove damaged extracellular matrix (ECM); throughout the proliferation phase, they collapse the capillary basement membrane, facilitating angiogenesis and cell migration; and during the remodelling phase, they contract and reconstruct skin tissue. All wounds require a threshold of these enzymes for optimal healing. However, high enzymatic activities can cause excessive breakdown and decreased wound healing [38]. For example, TGF-β has been demonstrated to induce senescence and senescence-related characteristics in fibroblasts [39]. Similarly, MCP has been shown to be a SASP-related protein [40]. We found significantly elevated *Mmp3*, *Mmp9*, *Mcp*, and *TGF-β* mRNA levels in chronic wounds (Figure 1F), potentially contributing to the increased senescent cell abundance in chronic wounds.

### Elevated *p16^Ink4a^* and *p21^Waf1/Cip1^* expression in epidermal and dermal tissues from chronic compared to acute wounds

**Figure 2:**
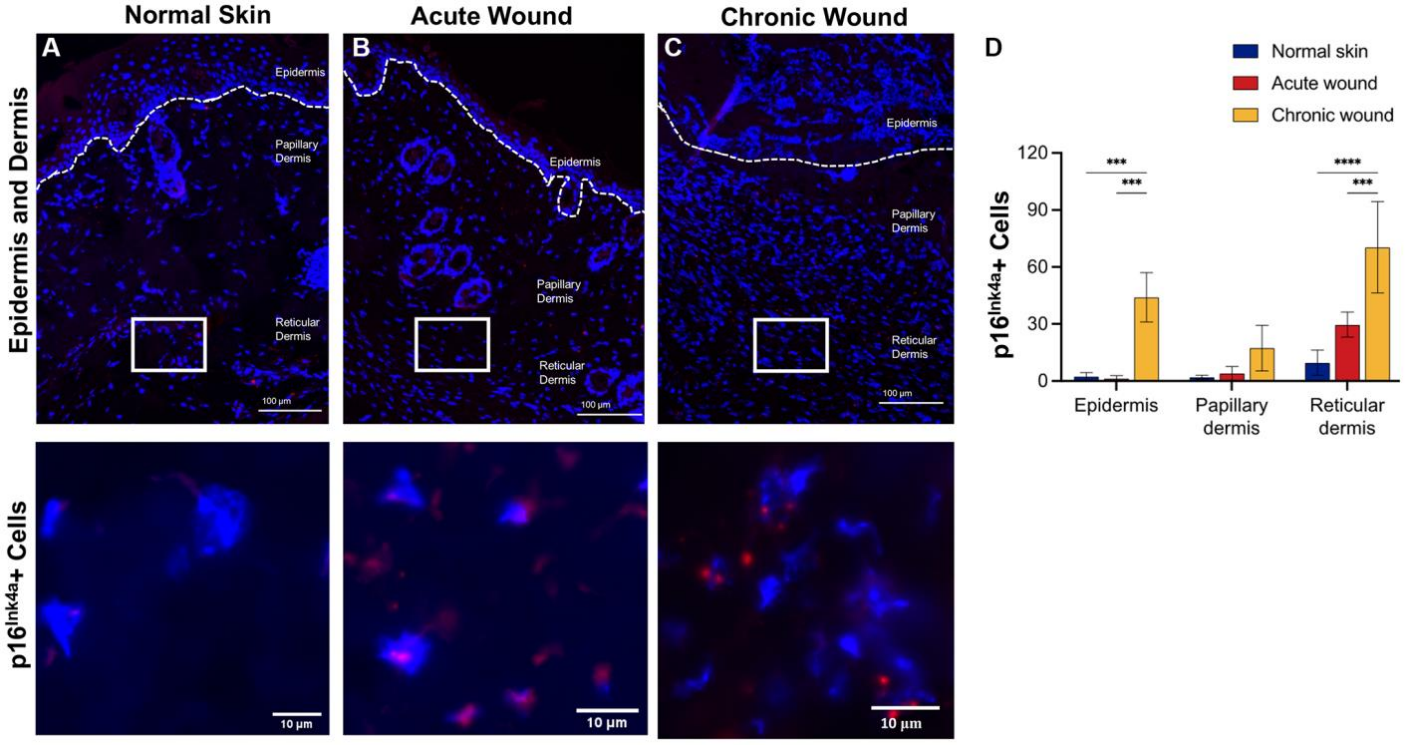
***p16^Ink4a^* RNA-ISH transcription is upregulated in dermal tissues in chronic wounds.** Representative RNA-ISH images showing *p16^Ink4a^* nuclear localization in (**A**) normal skin, (**B**) an acute wound, and (**C**) a chronic wound, 20× magnification (top) and focused zoom (bottom) (*n* = 6 in each group at day 14). (**D**) Quantification of *p16^Ink4a^* positive cells in epidermis, papillary dermis, and reticular dermis. Measurements are expressed as mean ± SEM. Statistical analysis was performed using Student’s *t*-test; ^***^p < 0.001, ^****^p < 0.0001. RNA-ISH, RNA *in situ* hybridization.

**Figure 3:**
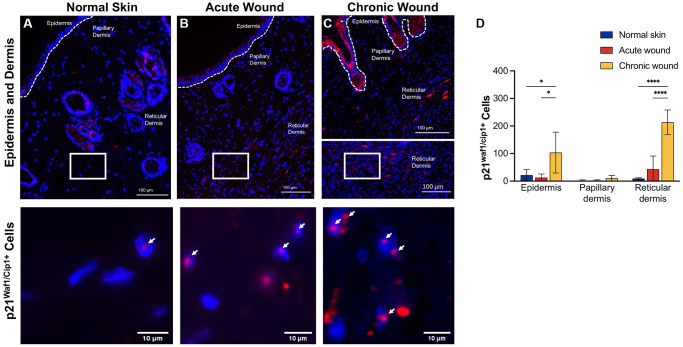
***p21^Waf1/Cip1^* RNA-ISH transcription is upregulated in both epidermal and dermal tissues in chronic wounds.** Representative RNA-ISH images showing *p21^Waf1/Cip1^* nuclear localization in (**A**) normal skin, (**B**) an acute wound, and (**C**) a chronic wound, 20× magnification (top) and focused zoom (bottom) (*n* = 6 in each group at day 14). (**D**) Quantification of *p21^Waf1/Cip1^* positive cells in epidermis, papillary dermis, and reticular dermis. Measurements are expressed as mean ± SEM. Statistical analysis was performed using Student’s *t*-test; ^*^p < 0.05, ^****^p < 0.0001. RNA-ISH, RNA *in situ* hybridization.

CDKs phosphorylate and modulate various proteins are involved in cell cycle progression. CDK inhibitors encoded in the *CDKN1A* (*p21^Waf1/Cip1^*), *CDKN2A* (*p16^Ink4a^*), and *CDKN2B* (*p15^Inc4b^*) loci are key drivers of cell cycle arrest in senescent cells [41] and have been used as markers of cellular senescence [13, 42, 43]. RNA *in situ* hybridization data evaluated *p16^Ink4a^* (Figure 2) and *p21^Waf1/Cip1^* (Figure 3) in the epidermis, papillary dermis, and reticular dermis. Low numbers of *p16^Ink4+^*-positive cells were identified in the epidermis and papillary dermis (Figure 2A–2C). There was a statistically significant higher number of *p16^Ink4a^* cells with 2–3 nuclear foci in chronic wounds (Figure 2D). In contrast, there were higher numbers of *p21*^Waf1/Cip1^-positive cells in the epidermis and reticular dermis of chronic wounds, with 2–3 prominent nuclear foci compared to acute wounds (Figure 3A–3C). This was statistically significantly higher in chronic wound beds compared to normal skin and acute wounds (Figure 3D).

## DISCUSSION

Cutaneous wound healing is a highly regulated process that is integral to maintaining the skin barrier. In acute wounds due to, for example, trauma, surgery, or even bug bites, a well-coordinated series of events are deployed for proper wound healing. In contrast, a chronic wound can form when pathologic factors are present such as poor blood supply (peripheral vascular disease), immune dysfunction (immunosuppression or acquired immunodeficiency), metabolic diseases (diabetes), medications, or prior local tissue injury (radiation therapy) [44]. A chronic wound is a wound that has deviated from its natural physiologic course into a stalled end point. It appears that the sequence of wound healing events involves an intricate interplay, which can be better understood when cellular senescence is appreciated as a core regulator of regeneration [45]. Here, we report a chronic wound healing model that can be used to decipher the paradoxical role of cellular senescence in acute versus chronic wound healing.

Temporal dynamics of senescent cells during wound healing and effects of senescent cell removal on acute wound repair have been reported [46, 47]. In an acute wound, eliminating p16- and p21-positive cells led to delayed wound closure. Conversely, we report that chronic wounds have higher expression of *p21^Waf1/Cip1^* in all skin layers compared to *p16^Ink4a^*, suggesting better specificity of p21 as a senescence marker in pathological chronic wounding. Despite their cell cycle arrest, persisting senescent cells in the wound bed remain metabolically active and communicate with their cellular environment through paracrine signaling, known as the SASP. Yet, transient senescence and the associated short-lived SASP were found to benefit the tissue repair environment [46]. This long-held dogma that senescent cells are beneficial when transiently present after acute injury, particularly in young tissue, has been challenged. Moiseeva et al. reported that senescence either transiently (in mild injury) or persistently (in chronic injury) was deleterious for muscle regeneration, irrespective of age [48]. Prior studies have shown that chronic wounds harbor senescent fibroblasts, which produce high levels of matrix-degrading proteases and inflammatory cytokines [49, 50]. Increased skin senescence and SASP markers in young mice were reported to be associated with delayed wound healing [51]. In accordance, we found increased expression of matrix metalloproteinases (*Mmp3* and *Mmp9*) and other proteases (*Mcp*) in the chronic murine skin wound, which could contribute to growth factor degradation and wound healing delay. As such, cellular senescence exhibits a dual action and context-dependent role in wound healing that appears to involve a continuum from a transient senescent cell-induced beneficial effect in acute wounding to a persistent senescent cell-induced detrimental effect in chronic wounds.

Our study has limitations. The oxidative stress-induced chronic wound bed was compared to the acute wound bed at a single timepoint. To adequately elucidate wound chronicity and its relation to cellular senescence, various timepoints from hours to days post-wounding will need to be examined in future studies. Nonetheless, these chronic wounds had impaired dermal-epidermal interactions, abnormal matrix deposition, and damaged vasculature, as is the case in human chronic wounds. Future studies will also need to evaluate sex differences, given biological differences. In addition, senescent cells are highly heterogeneous [52] and the markers we used in this study might not be specific to every type of senescent cell. More investigation is needed once more sensitive and specific senescence markers have been developed. Other limitations of murine models for wound healing include variations in wound contracture rate compared to human wounds that heal by granulation and epithelial cell resurfacing of granulation tissue [53].

Wound care specialists often encounter stalled wounds after they have reached chronicity for weeks or months, limiting understanding about the inciting event of such stalled wounds. The number of patients afflicted with chronic wounds has been growing annually since the prevalence of diabetes and other chronic diseases that impact on wound healing has been increasing [54]. Thus, a preclinical animal model that recapitulates the complexity of human chronic wounds holds high value. To our knowledge, this study is the first chronic wound murine model to profile the effects of the chronic cellular senescence that is linked to delayed wound healing. This may have implications for developing interventions that target cellular senescence for chronic or stalled wounds as a root cause-driven therapeutic strategy.

## Supplementary Materials

Supplementary Table 1
