# Supplementary material for: A chronic wound model to investigate skin cellular senescence
Source: Aging (Albany NY). 2023 Apr 21;15(8):2852–62. doi: 10.18632/aging.204667 (PMC10188333; doi:10.18632/aging.204667)
Supplement: Supplementary Table 1 [file aging-15-204667-s001.pdf]

SUPPLEMENTARY TABLE

Supplementary Table 1. RT-qPCR primers.

	RT-qPCR Primers (5′–3′)	
**Gene symbol**	**Forward**	**Reverse**
Mmp3	CTCTGGAACCTGAGACATCACC	AGGAGTCCTGAGAGATTTGCGC
Mmp9	GCTGACTACGATAAGGACGGCA	TAGTGGTGCAGGCAGAGTAGGA
IL-6	GTC AAC TGC ATG AAC AGA AAG G	AGC AGG CAG GTC TCA TTA TTC
Mcp	CATCACGGACAGAGGTTCTGAG	TCCTCTGTTGTGTGGATTCACTC
Tgf-β	GCCTGAGTGGCTGTCTTTTGA	CACAAGAGCAGTGAGCGCTGAA
Act-b	CATTGCTGACAGGATGCAGAAGG	TGCTGGAAGGTGGACAGTGAGG

Transcript levels were quantified using the $2^{\Delta\Delta Ct}$ method and normalized to the housekeeping gene β- actin, using gene specific primer sequences.
